# The Effect of Resveratrol on the Cardiovascular System from Molecular Mechanisms to Clinical Results

**DOI:** 10.3390/ijms221810152

**Published:** 2021-09-21

**Authors:** Roland Gal, Laszlo Deres, Kalman Toth, Robert Halmosi, Tamas Habon

**Affiliations:** 1 Division of Cardiology, 1st Department of Medicine, Medical School, University of Pecs, 7624 Pecs, Hungary; gal.roland@pte.hu (R.G.); deres.laszlo@pte.hu (L.D.); toth.kalman@pte.hu (K.T.); halmosi.robert@pte.hu (R.H.); 2 Szentágothai Research Centre, University of Pecs, 7624 Pecs, Hungary

**Keywords:** resveratrol, oxidative stress, inflammation, cardiovascular diseases, heart failure

## Abstract

Cardiovascular diseases are the leading causes of death worldwide. The cardioprotective effects of natural polyphenols such as resveratrol (3,5,4-trihydroxystilbene) have been extensively investigated throughout recent decades. Many studies of RES have focused on its favorable effects on pathological conditions related to cardiovascular diseases and their risk factors. The aim of this review was to summarize the wide beneficial effects of resveratrol on the cardiovascular system, including signal transduction pathways of cell longevity, energy metabolism of cardiomyocytes or cardiac remodeling, and its anti-inflammatory and antioxidant properties. In addition, this paper discusses the significant preclinical and human clinical trials of recent years with resveratrol on cardiovascular system. Finally, we present a short overview of antiviral and anti-inflammatory properties and possible future perspectives on RES against COVID-19 in cardiovascular diseases.

## 1. Introduction

Cardiovascular diseases (CVDs) are the leading causes of morbidity and mortality in developed countries and will lead to an expected worldwide number of CVD-related deaths of more than 23.6 million by 2030 [1,2]. According to the definition of the World Health Organization (WHO), CVDs are a group of disorders of the heart and/or blood vessels and include hypertension, coronary heart disease, cerebrovascular disease, peripheral arterial disease, cardiomyopathies, and heart failure [3]. A large portion of CVDs are caused by atherosclerosis, a degenerative process of the arterial wall that is triggered by chronic inflammation-induced oxidative stress. However, in the development of other cardiovascular diseases, such as heart failure, a complex interplay between metabolic and molecular alterations in oxidative stress, inflammation, lipid metabolism, and endothelial and myocardial dysfunction are crucial contributing factors [4,5].

The potential benefits of dietary interventions and nutrient supplementation in decreasing cardiovascular morbidity and mortality have been extensively investigated throughout recent decades. In the late 1980s, it gained worldwide attention that French people have a lower incidence of cardiovascular diseases and lower cardiovascular death rate despite having similar CV risk profile and similar saturated fat intake to people in other industrialized countries. This phenomenon is known as the French paradox and is attributed to higher red wine intake by the French [6]. Red wine contains high amount of polyphenolic compounds such as resveratrol (RES), catechin, and quercetin, and this is considered to be primarily responsible for the cardioprotective effect of red wine [7,8].

RES (3,5,4-trihydroxystilbene), a natural phytoalexin found in a wide variety of plants (e.g., nuts, berries, and grapes) is produced in response to environmental stress. RES exists as two geometric isomers: cis-(Z) and trans-(E). Trans- and cis-resveratrol can be either free or bound to glucose. The trans- form seems to have the major biological effects [9].

RES is well-absorbed in the jejunum and ileum after oral administration; however, its bioavailability is poor. Pharmacokinetic trials with trans-resveratrol have shown very low serum levels of unmetabolized RES after oral administration, [10] but after absorption, RES undergoes rapid and extensive metabolism in the intestine and liver, which is responsible for the low plasma levels [11,12]. However, according to the literature, the conjugate metabolites (resveratrol glucuronides and sulphates) also have relevant biological activities; furthermore, RES accumulates in several tissues [13,14,15]. In 2017, Böhmdorfer et al. demonstrated that in mice, the concentration of unconjugated RES in heart tissue (3.698 ± 2.519 nmol/g) was significantly (approximately 30×) higher compared to the plasma (0.140 ± 0.012 nmol/mL) level 30 min after administration. In addition, the concentration of its conjugated metabolites (e.g., resveratrol-3-O-glucuronide; 2.155 ± 1.284 nmol/g) was also approximately 10× higher in heart tissue 30 min after administration compared to plasma levels (0.289 ± 0.102 nmol/mL) [16]. Moreover, according to Biasutto et al., although low serum RES levels were detected in numerous human studies, up to 76% of RES is not accounted for if only plasma is analyzed. If the whole blood evaluation was performed, also including the cellular fraction, the validity of the measurement would be more representative [17]. Regardless of doses, the plasma half-life of RES in humans is generally 4–8 h [18].

Despite the several in vitro and in vivo positive trials with RES, controversial studies can also be found with this compound in the literature [19,20,21]. The unfavorable properties of RES mainly depend on duration of treatment, plasma concentration, or the type of cells or tissues [21,22,23,24,25,26]. A wide range of RES dose has been administered in different human and preclinical studies; however, the cutoff dose of toxicity is unknown. The most common adverse effects were gastrointestinal. Brown et al. found in humans that vomiting, diarrhea, and mild liver dysfunction may occur at doses of 2.5 g or more per day [27]. Furthermore, Crowell et al. published that high-dose (3 g/kg/day) oral supplementation of RES for 4 weeks resulted in nephrotoxicity (reported as elevated serum creatinine level or histopathological changes) in rats [20]. In a human study by la Porte et al., 2 g RES twice daily was well-tolerated by healthy subjects; however, diarrhea was frequently observed (six of the eight subjects) [28].

A large number of preclinical and human studies have demonstrated the beneficial effects of this bioactive polyphenol in cardiovascular diseases. The aim of this article is to summarize the protective, favorable effects of RES on the cardiovascular system. Moreover, we overview the relevant preclinical and human clinical trials of recent years, focusing on the connection between RES and the cardiovascular system.

## 2. Antioxidant Effects of RES

Oxidative stress has an important role in the pathogenesis of different cardiovascular pathologies, such as formation of atherosclerotic plaques, ischemia-reperfusion injury, cardiac remodeling (hypertrophy and fibrosis), and heart failure [29,30].

### 2.1. Exogenous Antioxidant Properties of RES

**Figure 1:**
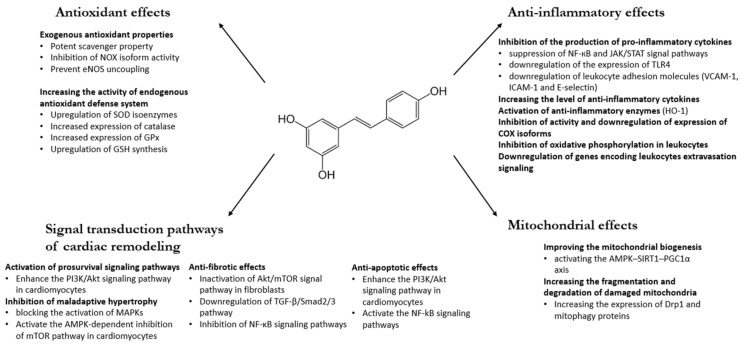
Beneficial effects of RES on cardiovascular system. Akt: protein kinase B; AMPK: AMP-activated protein kinase; COX: cyclooxygenase; Drp1: dynamin-related protein 1; GPx: glutathione peroxidase; GSH: glutathione; eNOS: endothelial nitric oxide synthase; HO-1: heme oxygenase-1; ICAM: intracellular cell adhesion molecule; JAK: Janus kinase; MAPK: mitogen-activated protein kinase; mTOR: mammalian target of rapamycin; NF-κB: nuclear factor kappa B; NOX: NADPH oxidases; PI3K: phosphatidylinositol 3-kinase; PGC-1α: peroxisome-proliferator-activated receptor gamma coactivator 1-alpha; SOD: superoxide dismutase; STAT: signal transducer and activator of transcription proteins; TGF-β: transforming growth factor; TLR4: toll-like receptor 4; VCAM: vascular cell adhesion molecule. References: [31,32,33,34,35,36,37,38,39,40,41,42,44,45,46,47,55,56,57,59,60,61,62,64,68,69,70,71,73,74,78,79,84,85,86,87,92,94,97,98,100,101,105].

Given that RES has a marked scavenger effect, it is not surprising that it can protect cells against oxidative damages. ROS-induced damage can be diminished by two factors: scavenging of free radicals and inhibiting the radical formation. As a polyphenolic compound, RES as an exogenous antioxidant has been shown in in vitro systems to directly scavenge free radicals, including superoxide (O_2_•^−^), hydroxyl radical (•OH), hydrogen-peroxide (H_2_O_2_), and peroxynitrite (Figure 1) [31,32,33]. Numerous in vitro studies with different tissues and cell cultures revealed the potent scavenger property of RES in recent years. In 2009, Wang et al. showed that RES prevented cisplatin-induced toxicity of rat cardiomyocytes via scavenging free radicals and via increasing the levels of antioxidants [34]. Leonard et al. demonstrated in murine macrophage cell culture that RES successfully scavenged hydroxyl, superoxide, and metal-induced radicals, thereby significantly decreasing lipid peroxidation and DNA damage [35]. Moreover, Ungvari et al. found that RES increased the oxidative stress resistance of endothelial cells and prevented them from oxidative stress-induced endothelial cell death via scavenging free radicals (H_2_O_2_) [36].

In a recent in vitro study, Hosoda et al. published that RES and its hydroxyl analog (piceatannol) successfully decreased hydrogen peroxide-induced injury in mouse muscle cells via its scavenging activity [37].

RES decreases oxidative stress not only by its scavenging property, but also via reducing ROS production. In cardiomyocytes and in endothelial cells, the NADPH oxidases (NOX isoforms) are important sources of ROS. RES can attenuate the activity of the NADPH oxidases via SIRT1-induced deacetylation of NF-κB [38].

Moreover, RES can prevent eNOS (endothelial nitric oxide synthase) uncoupling by upregulating GTP cyclohydrolase 1 (GCH1), which is a rate-limiting enzyme of tetrahydrobiopterin (BH4) biosynthesis. Major mechanisms underlying eNOS uncoupling under oxidative stress are the lack of the eNOS substrate l-arginine or the eNOS cofactor BH4 via their oxidation by potent oxidizing agents (ROS) [39,40].

These studies confirmed that RES, despite its low plasma concentration, is an effective antioxidant molecule thanks to its accumulation in the cardiac and vascular tissues and due to the formation of active metabolites.

### 2.2. The Effects of RES on the Endogenous Antioxidant Defense System

Beyond its scavenger effect, RES also has antioxidant effects in other ways. RES regulates the expression and activity of numerous antioxidant enzymes, thereby facilitating the elimination of free radicals.

In vivo and in vitro studies demonstrated the upregulation of superoxide dismutase (SOD) isoenzymes (SOD1 and SOD2) by RES [41], which is likely mediated by FOXO1 (Forkhead box O) transcription factor via its upregulation by SIRT1 (Figure 1) [42]. SIRT1 is probably also involved in the increased expression of catalase (CAT) and GP × 1 (glutathione peroxidase 1) caused by RES [43]. Moreover, in 2013, Khan et al. showed that both the expression and enzymatic activity of SOD2 were significantly upregulated by RES; however, RES only moderately upregulated the expression levels and activities of SOD1 and CAT enzymes [44]. Zhu et al. have also shown that the activity of the antioxidant system increased (SOD, GPx, and GSH) after RES treatment in mice liver tissue [45].

Many studies have suggested that Nrf-2 (nuclear respiratory factor) may be a major target of RES in relieving oxidative stress via activating AMPK/SIRT1/Nrf-2 associated antioxidant defense pathways. Nrf-2 acts as the master regulator of numerous genes encoding antioxidants and phase II-detoxifying enzymes (uridine-diphosphate-glucuronosyltransferases (UGTs)), glutathione S-transferases, sulfotransferases). Nrf-2 activation by RES also leads to an upregulation of γ-glutamylcysteine synthase, the rate-limiting enzyme for GSH (glutathione) synthesis. Consistently, RES can increase cellular GSH content [46,47].

## 3. Anti-Inflammatory Mechanism of RES

RES has been proved to have potent anti-inflammatory effect in many in vivo and in vitro studies [48,49,50,51].

According to several studies, one of the key functions of RES is to inhibit the production of pro-inflammatory cytokines such as tumor necrosis factor alpha (TNF-alpha) and interleukin-1β (IL-1β) and IL-6 [50,51,52].

Nuclear factor (NF)-κB and STAT proteins are important transcription factors in macrophages regulating physiological and inflammatory processes, as well as apoptosis. These signaling pathways can be activated by TLR4 (toll-like receptor 4—transmembrane protein of macrophages, which leads to the upregulation of the expression of pro-inflammatory mediators including IL-1β, IL-6, and TNF-α) [53,54]. According to recent studies, RES exerts anti-inflammatory effects by suppression of NF-кB and JAK/STAT (Janus kinase/signal transducers and activators of transcription) signal pathways [55,56]. Furthermore, Pinheiro et al. found that RES can inhibit the activation of monocytes via the induction of deacetylase (acetylated apurinic/apyrimidinic endonuclease-1/reduction-oxidation factor 1 (APE1/Ref-1)) and the reduction of demethylase activities (lysine-specific histone demethylase-1 (LSD1)) of key enzymes that mainly affect regulatory cascades (NF-кB and JAK/STAT mediated pathways) of transcription [56].

Moreover, Yang et al. demonstrated that RES can directly inhibit the expression of TLR4 [57]; hence, RES can be used for silencing TLR-mediated inflammatory responses and chronic diseases (cardiovascular diseases, obesity, or diabetes mellitus) linked to TLR activation [58].

In addition, RES can also silence inflammatory responses as an estrogen receptor (ER) ligand, predominantly mediated by the upregulation of SIRT1. The activation of SIRT1 by RES inhibits the RelA (member of NF-κB) acetylation, which in turn moderates the NF-κB-induced expression of inflammatory factors and the activation of leukocytes [50,59].

Zhang et al. provided a new insight into the anti-inflammatory effect RES. RES can cause downregulation of leukocyte adhesion molecules (VCAM-1, ICAM-1 and E-selectin) by the inhibition of TNF-α-induced NF-κB activation [60]. Moreover, RES can activate the Nrf-2 pathway, leading to decreased expression of ICAM-1 and then the inhibition of monocyte adhesion [61].

On the other hand, RES can increase the level of anti-inflammatory cytokines. In 2017, Palacz-Wrobel et al. published that RES, in a concentration-dependent manner (10–30 μM), causes an increase in gene expression and secretion of IL-10 from macrophages. Interestingly, this study also proved suppressive effect of RES on the production and secretion of the pro-inflammatory cytokine TNF-α by blocking its transcription [62].

Furthermore, RES induces deacetylation and activation of PGC-1α (peroxisome proliferator-activated receptor gamma coactivator 1-alpha) by activation of SIRT1. This is a very important mechanism, because under inflammatory conditions, PGC-1α level is downregulated, which decisively contributes to the enhanced inflammatory response [63].

RES exerts its favorable effects on inflammation not solely due to its influence on the production of various cytokines, but also via activating anti-inflammatory enzymes such as heme oxygenase-1 (HO-1), which is expressed in macrophages. Son et al. demonstrated that RES is able to induce the expression of the anti-inflammatory HO-1 via inhibiting the expression of TNF-α and IL-1β [64]. HO-1 is induced by oxidative or nitrosative stress, and it provides negative feedback for white blood cell activation, thereby significantly reducing inflammatory events including leukocyte adhesion and migration, as well as the production of pro-inflammatory cytokines [65]. In addition, HO-1 is a rate-limiting enzyme that degrades the pro-oxidant heme into carbon monoxide, free iron, and biliverdin that is rapidly converted into bilirubin [66]. RES can also suppress chronic vascular inflammation resulting in atherosclerotic plaque progression, by acting against pro-atherogenic oxysterol signaling in M1 (inflammation-encouraging) and M2 (inflammation-decreasing) macrophages [67].

Cyclooxygenase (COX) enzymes, which have two isoforms (COX-1 and COX-2), are key enzymes of prostaglandins biosynthesis; therefore, they have an important role in inflammatory processes. The antioxidant activity of RES and its ability to inhibit enzymes (COX-1 and COX-2) that are involved in the production of eicosanoids (PGE_2_, TXA_2_) can contribute to its marked anti-inflammatory (COX-2) and antiplatelet effect (COX-1) [68]. Moreover, RES can attenuate COX-2 expression via phosphorylation and acetylation of p65, c-Jun, and Fos transcription factors and can reduce their binding to the COX-2 promoter [69]. What is more, Yang et al. found that RES suppressed bradykinin-induced COX-2/PGE2 production via activation of SIRT1. SIRT1 can inhibit (acetylation) the AP-1 (activated by MAPKs) and NF-κB transcription factors, which are essential for COX-2 gene expression and for PGE2 release [70].

The anti-inflammatory effects of RES on the cardiovascular system have already been evaluated in some preclinical and in vitro studies (Figure 1), but data from human clinical studies are very limited.

In a recent human clinical trial, our workgroup showed that in RES-treated heart failure patients, the level of proinflammatory cytokines (IL-1, IL-6) decreased via inhibition of oxidative phosphorylation in leukocytes, which is directly proportional to their activity. Moreover, the mRNA profile analysis of leukocytes revealed a significant decrease in genes encoding leukocyte extravasation signaling and B cell receptor signaling [71]. 

## 4. Beneficial Effects of RES via Different Signal Transduction Pathways

### 4.1. Effect of RES on Pro-survival Signal Pathways

The PI3K (phosphatidylinositol 3-kinase)/Akt (protein kinase B) signaling pathway is a powerful pathway that promotes cell survival and growth in response to extracellular stress situations (e.g., oxidative stress). PI3K-mediated Akt activation can inhibit transcription factors that promote the expression of cell death genes, and it enhances the activity of FOXO transcription factors and therefore the transcription of anti-apoptotic, pro-survival genes [72].

RES further augments the ROS-induced activation of endogenous pro-survival signaling pathways, including the PI3K/Akt pathway. Akt activation by RES can inhibit ROS-mediated apoptosis and improves survival of cardiomyocytes [73] via Akt-mediated phosphorylation (inhibition) of the Bcl-2 family (regulator proteins of apoptosis) and GSK-3β [74].

Moreover, the PI3K/Akt signaling pathway is commonly involved in the Nrf-2-dependent transcription in diverse cell types of responding to ROS insults [75]. In a recent trial, Zhuang et al. demonstrated that RES can decrease ROS-induced damage via PI3K/Akt mediated Nrf-2 phosphorylation (activation) and can consequently increase antioxidant gene (SOD-1, CAT, GSH-Px) expression [76].

### 4.2. Anti-Fibrotic Effects of RES

Cardiac fibrosis is a key feature of cardiac remodeling, and it is characterized by the excessive deposition of extracellular matrix (ECM) by fibroblasts between myofibrils [77].

PTEN (phosphatase and tensin homolog) is known as a negative regulator protein of the PI3K/AKT/mTOR and TGF-β/Smad2/3 signaling pathways, which in turn are involved in the development of cardiac remodeling and fibrosis [77].

In 2019, Zou et al. found that a 2-week-long RES treatment significantly attenuated cardiac dysfunction and fibrosis via inhibiting PTEN degradation and thus inhibiting its downstream mediators (PTEN/Akt/Smad2/3 and NF-κB signaling pathways) in a pressure-overload-induced rat cardiac fibrosis model [78]. In addition, Chen et al. demonstrated that RES can moderate cardiac hypertrophy and fibrosis, inhibiting PTEN degradation via inactivation of Akt/mTOR (mammalian target of rapamycin) and activation of AMPK (AMP-activated protein kinase) signals [79].

TGF-β/Smad2/3 is a central signaling pathway in the regulation of ECM production and cardiac fibrosis. SIRT1 activation by RES attenuated isoproterenol-induced cardiac fibrosis (fibroblast proliferation and collagen secretion) by downregulating the TGF-β/Smad2/3 pathway via silencing miR-17 gene and overexpressing Smad7 [80,81].

### 4.3. Maladaptive Hypertrophy

Mitogen-activated protein kinase (MAPK) cascades are key signaling factors that control a wide variety of cellular processes such as proliferation, cell survival, and apoptosis under both normal and pathological conditions (e.g., oxidative stress). ERK1/2 (extracellular signal-regulated kinase) is a downstream kinase of the MAPK pathway and is located in the cytoplasm of unstimulated cells; however, if activated, ERK1/2 is transferred to the nucleus and regulates the activity of various transcription factors through phosphorylation [82]. Overactivation of the MAPK signal pathway by oxidative stress has been shown to be involved in pathological cardiac hypertrophy [83]; however, RES can block the ROS-mediated activation of MAPK/ERK1/2 [84] via the increased production of MAPK phosphatase-1 (MKP-1), which is the major negative regulator of MAPKs [85].

The serine/threonine-specific protein kinase mTOR plays a central role in the regulation of many cellular processes, such as cell growth, metabolism, proliferation, survival, transcription, apoptosis, and autophagy, as a downstream target of some signal pathways (AMPK, PI3K/Akt). Recently, many trials have reported that RES can activate the AMPK-dependent inhibition of mTOR pathway and can also reduce the expression of the mTOR signaling protein. Downregulation of mTOR can be beneficial in inhibiting atherosclerosis (proliferation of smooth muscle cells and endothelial cells), cardiac hypertrophy, and remodeling [86,87].

Furthermore, in 2015, Dolinsky et al. found that RES can dose-dependently inhibit various pro-hypertrophic signaling pathways and that RES has differential effects on the modification of these signaling cascades in response to pathological stimuli versus physiological stimuli [88].

### 4.4. Apoptosis

RES has antiapoptotic and cardioprotective effects due to its antioxidant activity [89], which decreases oxidative stress and therefore apoptosis as well as cell death. Moreover, RES can activate the elements of the endogenous antioxidant system (GSH, SOD, CAT) [90], and it has also beneficial effects on numerous signaling pathways having important roles in I/R injury, inflammation, and apoptosis [91,92]. This favorable effect of RES is mediated via the PI3K/AKT axis, as well as via NF-kB and p53 signaling and via decreasing the expression of IL-1, IL-6, TNF-a, and IL-18 in vivo [91]. Results from in vitro experiments show that RES protects cardiomyocytes from H2O2-induced apoptosis through the activation of sirtuins [93]. Sirtuins are histone deacetylases that have extensive effects on cellular homeostasis, cell survival, inflammatory processes (Foxo3a, p53, NF-kB), the antioxidant system, and energetics (SOD2, PGC-1alpha). SIRT3 protects cells from oxidative-stress-mediated cell death by activating NF-kB [94,95].

## 5. Effects of RES on Mitochondria

Mitochondria are the most important source of energy in cells. In the process of oxidative phosphorylation, the mitochondrial ETC (electron transport chain) oxidizes nutrients, thereby releasing the chemical energy stored within the nutrients in order to produce adenosine triphosphate (ATP) [96].

### 5.1. Biogenesis and Function

New mitochondria are produced in the process of mitochondrial biogenesis, which is necessary for adaptation to the energetic requirements of cells. Inhibition of NADPH oxidase by RES induces the phosphorylation of AMP-activated protein kinase (AMPK), thereby activating the AMPK–SIRT1–PGC1α axis, which is a major axis involved in mitochondrial biogenesis and in the regulation of cellular energy metabolism. AMPK activity declines with age; thus, energy homeostasis is progressively disturbed during the aging process. Declining sensitivity of AMPK activation provokes chronic low-grade inflammation, increases cellular stress, and therefore raises the risk for age-associated CVDs [97,98]. AMPK, as an essential energy sensor in cells, controls the activity of SIRT1 (NAD+-dependent protein deacetylase) by regulating the level of nicotinamide adenine dinucleotide (NAD+). An increase in NAD+ level induces further SIRT1 activation, which in turn provokes deacetylation and activation of peroxisome proliferator-activated receptor gamma coactivator 1-alpha (PGC-1α), thereby stimulating mitochondrial biogenesis via activation of Nrf-1 and Nrf-2 transcription factors. These transcription factors activate the expression of some key nuclear-encoded ETC proteins of mitochondria [63,99].

Csiszar, through examining aortas of type 2 diabetic mice, has proved that RES induced mitochondrial biogenesis in endothelial cells by upregulation of SIRT1 and via a pathway that involves activation of eNOS and induction of mitochondrial biogenesis factors (including PGC-1α, Nrf-1) [100].

Furthermore, in 2017, Ma et al. also demonstrated the protective role of RES-activated SIRT1 in mitochondrial biogenesis and function in a diabetic mice cardiomyopathy model, through PGC-1α-mediated expression of mitochondrial regulatory genes of Nrf-1, Nrf-2, estrogen-related receptor-α (ERR-α), and mitochondrial transcription factor A (TFAM) [101].

### 5.2. Mitochondrial Dynamics

Mitochondria are highly dynamic organelles undergoing coordinated cycles of fission and fusion. These rapid and transient morphological adaptations are crucial for many cellular processes (cell cycle, immunity, mitochondrial quality control, and apoptosis); therefore, disorders of dynamics can lead to numerous human diseases [102]. Cardiomyocytes are particularly vulnerable to ischemia due to their high-energy demand and due to the lack of storage. The fragmentation of mitochondria was also observed in ischemic cardiomyocytes. Mitochondrial fission is regulated by dynamin-related protein 1 (Drp1), which binds to its specific receptors on the outer mitochondrial membrane for the promotion of mitochondrial fission processes [103,104]. In 2017, Ren et al. found that in cardiomyocytes (H9C2 cells), RES enhanced the expression of Drp1 and mitophagy proteins, suggesting that RES increased mitochondrial fragmentation and mitophagy removing damaged cardiac mitochondria [105].

## 6. Effects of RES on Endothelial Cells

Endothelial cells are key regulators of blood pressure, vascular tone, and hemostasis, and therefore play an essential and dynamic role in the cardiovascular system. The endothelium synthesizes important vasoactive substances, including prostacyclin, NO, and the vasoconstrictive endothelin-1 (ET-1). Endothelial NO reaches the smooth muscle cells (SMC) by diffusion and causes vasodilation [106,107]. Endothelial and vascular function can be improved by RES treatment via decreasing cholesterol and triglyceride levels and hence increasing nitrogen oxide (NO) levels. In this regard, the anti-inflammatory effect of RES can also have a role [108,109,110].

RES increases the production of nitric oxide (NO) in endothelial cells by upregulating the expression of endothelial NO synthase (eNOS), stimulating the enzymatic activity of eNOS, and preventing eNOS uncoupling [108,111]. RES-mediated activation of SIRT1 stimulates eNOS activity through deacetylation and enhances eNOS expression by deacetylation of Forkhead box O (FOXO) transcription factors (regulating the expression of genes involved in cell growth, proliferation, differentiation, and longevity) [112].

In addition, RES can reduce inflammatory cytokine-induced ET-1 synthesis in cultured endothelial cells by inhibition of ET-1 mRNA expression through attenuating the activator protein-1 binding site (AP-1) of the ET-1 promoter [113,114].

## 7. Preclinical and Clinical Studies with RES on Cardiovascular System

### 7.1. Atherosclerosis and Risk Factors of Cardiovascular Diseases

Atherosclerosis predominantly involves the intimal layer of the arterial vessel wall and is characterized by the deposition of lipids, the proliferation and migration of local smooth muscle cells, and chronic inflammation. It leads to luminal narrowing and/or thrombus formation, resulting in clinical events such as coronary artery disease, peripheral arterial disease, and stroke.

#### 7.1.1. Preclinical Evidence

The cardiovascular protective effects of RES are mainly based on its capabilities of reducing oxidative stress, moderating inflammation, and favorably modifying CV risk factors.

Deng et al. found that RES can decrease vascular inflammatory injury and atherogenesis via downregulating NF-κB p65 and p38 MAPK expression and upregulating SIRT1 expression [115]. The role of abnormalities in the cholesterol and lipoprotein metabolism is well-known in the pathogenesis of atherosclerosis. LDL exposed to the macrophages of an atherosclerotic lesion become oxidized. The oxidized LDL particles (LDL-ox) are able to damage endothelial cells, contributing to the progression of atherosclerotic lesions. Some preclinical studies have shown that RES could modify this process, notably by decreasing plasma triglyceride and LDL-cholesterol levels and by increasing HDL-cholesterol. In in vitro studies, RES inhibits the LDL and HDL oxidation [116] and can reduce the plasma oxidized LDL cholesterol level [117].

According to several animal studies in recent decades, RES supplementation reduces the level of triglyceride, total cholesterol, and LDL-C [118,119,120,121,122,123] via increasing the synthesis and efflux of bile acids [124] and moderating oxidative stress and inflammation [125,126]. In a recent experimental study from 2020, RES (20 mg/kg for 10 weeks) beneficially influenced the cholesterol concentrations in diabetic rats [127].

The cytokine IL-8, as well as the adhesion molecules VCAM and ICAM, together with the passive lipid accumulation in the arterial walls, are known to play an important role in the initiation of atherosclerosis [128,129].

Diabetes mellitus is also a major risk factor of cardiovascular diseases. According to experimental studies, RES can regulate glucose homeostasis by improving insulin sensitivity [130,131]. RES improves diabetic complications and restores glucose homeostasis via modulating SIRT1/AMPK/NF-κB [132,133] and p38-MAPK/TGF-beta1 [134] signaling pathways, inhibiting Drp1-mediated mitochondrial fission, and preventing ER stress-associated NLRP3 inflammasome activation [135].

Endothelial dysfunction induces atheromatous plaque formation and it is considered to be an important factor in the development of cardiovascular diseases [136]. Several studies have shown the favorable effects of RES on endothelial function in recent years.

RES can also effectively improve cardiac and vascular autonomic function, protecting erythrocytes via interacting with hemoglobin and reducing heme-iron oxidation [137].

#### 7.1.2. Human Clinical Trials

There are only a few human clinical trials with RES in CVD; however, RES appears to have a significant therapeutic potential against CVDs. The relevant randomized human clinical trials are summarized in Table 1.

In a human clinical trial, 400 mg trans-resveratrol was administered to 44 healthy participants for 30 days. After the termination of treatment, the blood plasma of participants was incubated with cultured human coronary artery endothelial cells. According to the results, a significant reduction in the messenger RNA expression of IL-8, VCAM, and ICAM was found due to RES treatment compared to the baseline plasma, and an inverse relationship was observed between the concentration of plasma RES and the expression of IL-8, VCAM, and ICAM. This study firstly proved that RES may have protective effects against atherosclerosis in low-CV-risk individuals, thereby suggesting that RES should receive consideration as a primary preventive agent [129,138].

Multiple potential mechanisms of RES to normalize the lipid profile in humans have already been examined. These include a decrease in mRNA expression of hepatic HMG-CoA (3-hydroxy-3-methyl-glutaryl-CoA) reductase and activation of SIRT1 [139], which may potentially lead to reverse cholesterol transport [140] and favorable alteration in lipid profile [141].

In parallel to the aforementioned studies, Simental-Mendia et al. examined the effect of RES in patients with newly diagnosed dyslipidemia. In total, 71 patients were enrolled in this randomized double-blind, placebo-controlled trial and were randomly allocated to receive either 100 mg/day RES or placebo. RES supplementation after 2 months significantly reduced total cholesterol and triacylglycerol concentrations in individuals with dyslipidemia [142].

In 2012, Magyar et al. found that RES administration (10 mg/day for 3 months, *n* = 40) significantly decreased the LDL-cholesterol levels in patients with ischemic heart disease, but no significant effect was detected on other lipid parameters such as total cholesterol, HDL cholesterol, and triglyceride levels [143]. In addition, our workgroup demonstrated in 2020 that in patients with heart failure, higher-dose RES (100 mg/day) for 3 months significantly decreased both LDL-cholesterol and total cholesterol levels; however, the HDL-cholesterol level was also slightly lower in the RES group compared to the placebo group [71].

However, in 2016, Bo et al. found that RES administration (500 mg/day for 24 weeks; *n* = 120) significantly increased the total cholesterol level, but LDL-cholesterol, HDL-cholesterol, and triglyceride levels did not change significantly in the RES group [144].

A meta-analysis by Asgary et al. showed that although RES did not significantly affect the total cholesterol level, HDL cholesterol level could be increased by RES treatment [145].

### 7.2. Vascular Function and Hypertension

#### 7.2.1. Preclinical Studies

RES administration could reduce blood pressure in several experimental models of hypertension, including angiotensin II- (146 mg/day for 4 weeks) [146], renal artery clipping (5, 10, or 20 mg/kg/day for 4 weeks) [147], hypoxia-induced hypertensive rats (10 mg/day for 4 weeks), and fructose-fed rats [148]. Several mechanisms are involved in the antihypertensive effect of RES, including AMPK phosphorylation, SIRT1 activation, increased NO levels, and decreased ROS production due to regulating NADPH oxidase, SOD-2, and glutathione reductase [146,147,148,149]. Franco et al. demonstrated that RES (30 mg/kg/day for 30 days) could mitigate oxidative stress (RES normalized the SOD level in treated group) and hypertension in adult obese rats [150]. According to another experimental model by Tain et al., RES can prevent high-fructose-intake-induced hypertension in adult rats via targeting oxidative stress, nutrient-sensing signals, and gut microbiota dysbiosis [151].

In a recent remarkable study by Prysyazhna et al., RES also decreased the blood pressure in hypertensive mice; however, a paradoxical mechanism of action of RES has been described. According to this new paradigm, RES can surprisingly induce direct protein oxidation, thereby activating cGMP-dependent PKG1α (protein kinase 1α), which can lead to vasorelaxation. This paradoxical, pro-oxidative property of RES is likely due to ROS formation upon electron transfer from RES to oxygen, especially during times of oxidative stress (diseased tissues), thus mediating beneficial signaling at the site of injury [152].

#### 7.2.2. Human Clinical Trials

Many clinical trials have reported that RES decreases blood pressure in patients with obesity [153], type 2 diabetes mellitus [154,155], or fatty liver disease [156]. Moreover, several meta-analyses indicate that RES intake reduces systolic and diastolic blood pressure at doses higher than 150 mg/day [157,158]. However, other meta-analyses of human clinical trials showed no significant effect of RES treatment on systolic and/or diastolic blood pressure [159]. In summary, beneficial effects of RES on blood pressure can only be observed when the effect is analyzed in obesity or in diabetes.

RES is known to improve endothelial function in animals; however, clinical trials are limited. Flow-mediated dilation (FMD) of the brachial artery is an important marker of endothelial function, and it can be used as an indicator of structural and functional endothelium changes. Wong et al. found in a pilot study that administration of RES (270 mg/day for 4 weeks) significantly increased FMD in overweight, hypertensive participants [160].

In 2012, Magyar et al. examined vascular function after 10 mg per day RES administration for 3 months in patients with stable coronary artery disease. Endothelial function was measured by FMD, and a significant improvement was found with regard to vasorelaxation in the RES-treated group compared to placebo [143].

In 2018, Marques et al. examined the effect of a single dose of trans-resveratrol (300 mg) on endothelial dysfunction in hypertensive patients. They proved that acute supplementation of trans-resveratrol caused an improvement in endothelial function measured by FMD; however, brachial blood pressure and aortic systolic blood pressure did not change significantly [161].

Red blood cell (RBC) aggregation and deformability significantly influences blood flow in coronary microcirculation. In 2020, Gal et al. demonstrated in a human clinical trial that RES (100 mg pro day for 3 months)-induced improvement of red blood cell aggregation may positively influence microcirculation, tissue perfusion, and oxygen supply, and hence can cause improvement in coronary and peripheral blood flow [162].

**Table 1:** Cardioprotective effects of resveratrol: relevant randomized human clinical trials and meta-analyses.

Area of Interest	Study Authors	Date	Type of Study	Study Design	Dose and Treatment Period of Resveratrol	Summary of Main Findings	Ref.
Atherosclerosis and Risk Factors							
Lipid profile	Magyar et el.	2012	RCT	40 patients with CAD	10 mg daily for 3 months	RES significantly decreased LDL-cholesterol levels, but no significant effect was detected on other lipid parameters	[143]
DM	Bhatt et al.	2012	RCT	62 patients with DM	250 mg daily for 3 months	RES improved glycaemic control	[153]
Atherosclerosis	Agarwal et al.	2013	RCT	44 healthy people	400 mg daily for 30 days	RES significantly decreased IL-8, ICAM, VCAM levels. RES may have protective effects against development of atherosclerosis in low-CV-risk individuals	[130]
Metabolic status	Hoseini et al.	2019	RCT	56 patients with CAD and DM	500 mg daily for 4 weeks	RES had beneficial effects on HDL-cholesterol levels, total-/HDL-cholesterol ratio, and glycemic control	[138]
Lipid profile	Simental-Mendia et al.	2019	RCT	71 patients with newly diagnosed dyslipidemia	100 mg daily for 2 months	RES significantly reduced total cholesterol level	[139]
Lipid profile	Gal et al.	2020	RCT	60 patients with HFrEF	100 mg daily for 3 months	RES significantly reduced total cholesterol and LDL-cholesterol levels	[71]
Lipid profile	Bo et al.	2016	RCT	120 patients with DM	500 mg daily for 24 weeks	Total cholesterol level increased significantly, but LDL-cholesterol, HDL-cholesterol, and triglyceride level did not change significantly in RES group	[144]
Lipid profile	Asgary et el.	2019	Meta-analysis	396 subjects	100–3000 mg daily	RES did not significantly affect TC level, but HDL-C level could be increased	[145]
Vascular Function and Hypertension							
Blood pressure	Timmers et al.	2011	RCT	11 healthy, obese men	150 mg daily for 30 days	RES significantly reduced systolic blood pressure	[153]
Blood pressure	Bhatt et al.	2012	RCT	62 patients with type 2 DM	250 mg daily for 3 months	RES improved systolic blood pressure	[154]
Blood pressure	Liu et al.	2015	Meta-analysis	247 subjects	more than 150 mg daily	RES significantly decreased systolic blood pressure level	[157]
Blood pressure	Fogacci et al.	2018	Meta-analysis	681 obese subjects	more than 300 mg daily	RES significantly decreased the systolic blood pressure	[158]
Endothelial function	Wong et al.	2011	RCT	19 overweight men or postmenopausal women with hypertension	single dose (270 mg)	RES significantly increased the FMD after acute supplementation	[160]
Endothelial function	Magyar et el.	2012	RCT	40 patients with CAD	10 mg daily for 3 months	RES induced significant improvement of FMD	[143]
Endothelial function	Marques et al.	2018	RCT	24 subjects with hypertension	single dose (300 mg)	RES significantly increased the FMD in women after acute supplementation	[161]
Arterial stiffness	Imamura et al.	2017	RCT	50 patients with type 2 DM	100 mg daily for 12 weeks	RES supplementation may improve arterial stiffness	[155]
Heart Failure							
Heart failure with reduced ejection fraction	Gal et al.	2020	RCT	60 patients with HFrEF	100 mg daily for 3 months	RES improved several parameters of heart function (systolic and diastolic function) as well as cardiac biomarker levels, exercise tolerance, and QoL	[71]

### 7.3. Heart Failure

Heart failure (HF) is a complex multifactorial condition caused by structural and/or functional cardiac abnormalities, resulting in a reduced cardiac output or elevated intracardiac pressure [163].

#### 7.3.1. The Effect of RES in Heart Failure—Preclinical Trials

There is a large amount of evidence that oxidative stress and the consequent chronic inflammation play an important role in the development of heart failure [164,165]. Oxidative stress activates different intracellular signaling pathways regulating cardiac remodeling, hypertrophy, survival of myocytes, apoptosis, and necrosis [29].

The antioxidant and anti-inflammatory properties of RES were intensively investigated in recent decades in various heart failure models in rodents. According to the literature, RES can improve the diastolic and systolic function of the heart, it can reduce atrial and left ventricular remodeling, and it can improve cardiac energetics, which may contribute to the cardioprotective effects of RES in heart failure. 

Ahmet et al. published a long-term (10 months) study in 2017 and found that RES supplementation (5 mg/kg/day) can significantly improve LV systolic function in a postinfarction (surgical ligation of left coronary artery) heart failure rat model [166]. In 2017, Riba et al. also investigated the effects of RES in a postinfarction heart failure rat model, where isoproterenol was used to induce myocardial infarction and postinfarction remodeling. According to the results, systolic left ventricular function was significantly increased, whereas plasma BNP levels, left ventricular wall thickness and dimensions were decreased after 8 weeks RES treatment (15 mg/kg/day). Moreover, RES moderated oxidative stress and favorably modified the activity of several intracellular signaling pathways (Akt-1/GSK-3β, p38-MAPK, ERK1/2, MKP-1, COX-2, and iNOS uncoupling). In summary, RES was capable of preserving LV function and moderated the severity of heart failure already after 2 months [167].

In another postinfarction heart failure rat model, Matsumura et al. in 2018 used cardiotoxic hydroxyeicosatetraenoic acid (HETE) to induce myocardial infarction and heart failure. The results showed that RES treatment (5.82 mg/kg/day) significantly improved the ejection fraction in rats and reduced postinfarction left ventricular and atrial remodeling. Interestingly, the mechanism by which low dose RES exerts its cardioprotective effects was independent of the classical SIRT1 and antioxidant pathways, suggesting another additional pathway [168].

Sung et al. investigated the effects of RES on cardiac structure and function in a pressure-overload–induced heart failure mice model. At the end of the study, RES improved diastolic function and reduced the LV diameters and volumes. RES treatment (150 mg/kg/day) also reduced cardiac fibrosis, hypertrophy, and remodeling via its antifibrotic and anti-inflammatory effects. However, systolic function did not change after a 2-week-long RES supplementation [169].

This result is in accordance with other preclinical trials. Wojciechowski et al. found that low dose RES supplementation (2.5 mg/kg/day for 28 days) regressed the pressure-overload-induced cardiac hypertrophy and remodeling in rats. RES also significantly reduced oxidative stress in cardiac tissue [170].

In a recent study, Ma et al. demonstrated that RES (25 mg/kg/day) reduces the development of myocardial hypertrophy and fibrosis via SIRT1 activation in mice with heart failure. The enhanced SIRT1 improved mitochondrial function through the deacetylation (activation) of PGC-1α and thereby the regulation of downstream proteins such as Nrf-1 and Nrf-2 [101]. Similar results were published by Bagul et al. In this study, RES (10 mg/kg/day for 8 weeks) decreased the cardiac hypertrophy in diabetic rats via its SIRT1-mediated antioxidant effects [38].

Not only heart function and remodeling but also exercise capacity showed significant improvement in several preclinical studies. Sung et al. demonstrated that RES supplementation (450 mg/kg/day for 2 weeks) may effectively improve fatigue and exercise intolerance in pressure-overload-induced heart failure mice, associated with favorably changed gut microbiota composition and increased whole-body glucose utilization [171]. According to a study by Hart et al., RES supplementation (100 mg/kg/day) for 12 weeks significantly enhances the exercise capacity of rats. This beneficial effect is mediated by enhanced mitochondrial biogenesis with the activation of the AMPK-SIRT1-PGC-1α pathway [172].

In addition to the aforementioned, the cardioprotective effects of RES in heart failure have also been evaluated in many other preclinical studies in recent years, including pressure overload [170,173], hypertension [174], diabetes [175], chemotherapy [176,177], and myocarditis-induced [178] as well as genetic models of heart failure [179].

#### 7.3.2. Human Clinical Trials with RES in Heart Failure

Despite the large number of preclinical studies that have reported the beneficial effects of RES in heart failure, the number of human clinical studies investigating these effects are very limited.

In a previous study Magyar et al. examined the possible cardioprotective effects of RES in patients after myocardial infarction with preserved ejection fraction (54.77 ± 1.64% at the baseline in the treated group) and found that the diastolic function was significantly improved after 10 mg/day RES administration for 3 months. However, the systolic function did not change significantly [143].

In 2013, Militaru et al. demonstrated that 20 mg RES per day administered for 2 months resulted in a significant decrease in NT-proBNP level (biomarker of heart failure) in patients with angina pectoris, but without proven heart failure [180]. These aforementioned studies were not designed specifically for heart failure.

In 2020, our workgroup firstly proved that RES beneficially influences heart failure in a randomized double-blind human clinical trial (RCT). A total of 60 outpatients with systolic heart failure in NYHA class II-III were enrolled in this clinical trial and randomized into two groups: receiving either daily 100 mg RES or placebo for three months. The RES treatment improved several parameters of heart function, including systolic and diastolic function, as well as global longitudinal strain. In parallel, the level of cardiac biomarkers of heart failure and remodeling (NT-proBNP and galectin-3) decreased significantly, and exercise tolerance as well as quality of life improved in the treated group. Moreover, RES exerted an anti-inflammatory effect measured by the decrease in levels of inflammatory cytokines (IL-1 and IL-6). According to the results, the decreased activity of leukocytes can be an important mechanism of RES, and it can contribute to its cardioprotective effect in heart failure [71].

## 8. Conclusions

RES is one of the most widely investigated polyphenolic compounds in cardiovascular diseases. In our review, we discussed the results of relevant preclinical and clinical trials of recent years of RES on cardiovascular diseases. The benefits of RES appeared to be mainly dependent on its antioxidant and anti-inflammatory properties, as well as the regulation of several signal transduction pathways of cell metabolism and longevity.

The discussed experiments confirmed that RES is an effective antioxidant agent thanks to its accumulation in the cardiac and vascular tissues and due to the formation of active metabolites. RES can effectively eliminate free radicals due to its marked scavenger effect and the ability to regulate the expression and activity of numerous antioxidant enzymes. RES exerts its favorable effects on inflammation not solely due to its influence on the production of various cytokines, but it can also regulate the activation of leukocytes and downregulate the activation of leukocyte adhesion molecules or COX isoforms. Moreover, RES influences different signal transduction pathways, mainly due to the activation of SIRT1; thereby, it can improve the survival of the cardiomyocytes as well as inhibit ROS-mediated maladaptive hypertrophy, apoptosis, and fibrosis.

Based on several preclinical and clinical trials, RES can positively modify CV risk factors (hypertension, diabetes mellitus, and lipid profile) and endothelial function, and it thereby can delay the progression of atherosclerosis and improve vascular function. Finally, several animal trials proved that RES beneficially influences heart failure, but the number of human clinical studies investigating these effects is very limited.

Despite numerous studies, the exact mechanisms of RES are still unclear and partly controversial, and its positive and protective effects in cardiovascular diseases have not been obviously confirmed in humans. In the future, additional preclinical trials are needed to understand the exact biochemical and cellular mechanisms, and more importantly, further adequately powered randomized, controlled human clinical trials with long-term follow-up periods are required to prove the efficacy of RES treatment of cardiovascular diseases.

## 9. Future Perspectives on Treatment with Resveratrol in COVID-19 Associated with CVDs

Based on the results of recent years, CVDs seem to be associated with higher morbidity and mortality in patients with COVID-19 infection. Myocardial and vascular injury in chronic cardiovascular diseases are strongly associated with SARS-CoV-2 infection, possibly due to the overactivation of inflammatory processes, namely due to hypercytokinemia (“cytokine storm”) triggered by viral infection [181]. Systemic inflammation and hypercoagulability results in myocardial damage, with consequent CV complications including myocarditis, myocardial infarction, heart failure, arrythmias, or venous thromboembolism (deep vein thrombosis or pulmonary embolism) [182,183,184,185]. RES has shown a high antiviral potential that can be explored in both human and animal viral infections. The antiviral mechanisms and effects of RES (inhibition of viral protein synthesis and some gene expressions) have been widely studied in various viruses, including coronavirus [186,187]. Moreover, RES can influence several mechanisms associated with CVD related to COVID-19, including upregulation of the expression of angiotensin-converting enzyme 2 (ACE2) [188]. The downregulation of ACE2 protein by SARS-CoV-2 is leading to dysfunction of the renin-angiotensin-aldosterone-system (RAAS), with overproduction of pro-inflammatory and pro-oxidant agents [189]. Furthermore, as a potent anti-inflammatory and antiviral agent, RES can inhibit the overactivation of pro-inflammatory responses as well as enhance the antiviral immune system (macrophages, cytotoxic T lymphocytes, and natural killer immune cells) [187].

In addition, based on its anti-thrombotic effects, RES can reduce the risk of vascular thrombotic events in the course of COVID-19 infection [190].

Although no preclinical or human clinical trials with RES have been published to date in COVID-19, the aforementioned mechanisms of action of RES suggest that it may contribute to the prevention of cardiovascular toxicity in COVID-19. Thus, in the future, RES may be an adjunctive agent in the treatment of SARS-CoV-2 infection.
